# N95 Filtering Facepiece Respirator Reuse, Extended Use, and Filtration Efficiency

**DOI:** 10.1001/jamanetworkopen.2024.41663

**Published:** 2024-10-29

**Authors:** Ralph C. Wang, Newton Addo, Nida F. Degesys, Jahan Fahimi, James S. Ford, Efrat Rosenthal, Anna R. Harris, Anna Q. Yaffee, Susan Peterson, Richard E. Rothmann, John DeAngelis, Vaishal Tolia, Manish N. Shah, Thomas B. Stephenson, Sheila J. Nogueira-Prewitt, Katherine N. Yoon, Edward M. Fisher, Maria C. Raven

**Affiliations:** 1 Department of Emergency Medicine, University of California, San Francisco; 2 Department of Emergency Medicine, Emory University, Atlanta, Georgia; 3 Department of Emergency Medicine, Johns Hopkins University, Baltimore, Maryland; 4 Department of Emergency Medicine, University of Rochester School of Medicine and Dentistry, Rochester, New York; 5 Department of Emergency Medicine, University of California, San Diego; 6 BerbeeWalsh Department of Emergency Medicine, University of Wisconsin-Madison; 7 Applied Research Associates, Engineering Science Division, Respiratory Protection Center of Excellence, Panama City, Florida; 8 Centers for Disease Control and Prevention, Atlanta, Georgia; 9 National Institute for Occupational Safety and Health, Washington, DC; 10 National Personal Protective Technology Laboratory, Pittsburgh, Pennsylvania; 11 Philip R. Lee Institute for Health Policy Studies, University of California, San Francisco

## Abstract

This cohort study examines the association of reuse of N95 filtering facepiece respirators and N95 filtration efficiency.

## Introduction

N95 filtering facepiece respirators (N95s) are designed to filter 95% or more of viral particles and prevent aerosolized transmission, reducing respiratory viral infections among health care clinicians.^1^ Under routine circumstances, N95s are discarded after 1 patient contact.^2^ During the COVID-19 pandemic, the Centers for Disease Control and Prevention provided strategies for extended use and limited reuse to manage shortages. Emergency department (ED) workers practiced a combination of both, which we termed reuse.^3,4,5^ Evidence assessing the impact of reuse on filtration efficiency (FE) in clinical settings is needed to inform conservation practices during N95 shortages. We assessed the association of reuse with N95 FE during the COVID-19 pandemic. We hypothesized that N95s reused for an increasing number of clinical shifts would result in reductions in FE, which would differ by respirator model.

## Methods

The Western Copernicus Group institutional review board approved this cohort study. We adhered to the Strengthening the Reporting of Observational Studies in Epidemiology (STROBE) reporting guideline.

We conducted a multicenter, prospective cohort study at 6 United States EDs to assess N95 reuse by physicians, nurse practitioners, and nurses on N95 performance and safety (including FE) from April 2021 to July 2022.^6^ Respirator models were grouped into 3M1860 (respirator 1), 3M1870 (respirator 2), and Halyard (respirator 3) categories. The primary outcome was FE and reduced FE (below 95%) (eAppendix in Supplement 1). The exposure of interest was reuse, defined based on number of shifts (1, 2, 3, 4, or 5) worn. We reported mean and median FE by shifts worn, stratified by N95 categories, and assessed differences in FE by shift using analysis of variance (ANOVA). A mixed-effect model (including number of shifts used, N95 model, use of skin protectant, and donning and doffing) assessed factors associated with reduced FE. We performed analyses using R version 4.4.1 (R Project for Statistical Computing) with significance set at *P* < .05. All tests were 2-sided. Data were analyzed from June 2023 to February 2024.

## Results

We enrolled 365 participants with N95s assessed for FE. Of these, 188 (52%) were physicians, 221 (61%) were female, and the median (IQR) age was 34 (30-41) years. Additionally, 148 respirators (40.5%) were respirator 1, 119 (32.6%) were respirator 2, and 98 (26.8%) were respirator 3. Wearing N95s for an increasing number of shifts was associated with reduced mean FE (ANOVA *P* value <.001) (Figure). The proportion of respirators with FE less than 95% increased when they were worn for more shifts—1.8% (95% CI, 0.4%-5.0%) after 1 shift; 10.1% (95% CI, 4.5%-17%) after 2 shifts; and 28.8% (95% CI, 18%-43%) after 3 shifts (Table). The adjusted odd ratio (OR) for reduced FE for 2 shifts of wear was 5.68 (95% CI, 1.43-22.5) and 35.4 (95% CI, 8.67-145) for 3 shifts of wear as compared with 1 shift a wear. The adjusted OR for Halyard was 9.84 (95% CI, 3.76-27.7).

**Figure.**
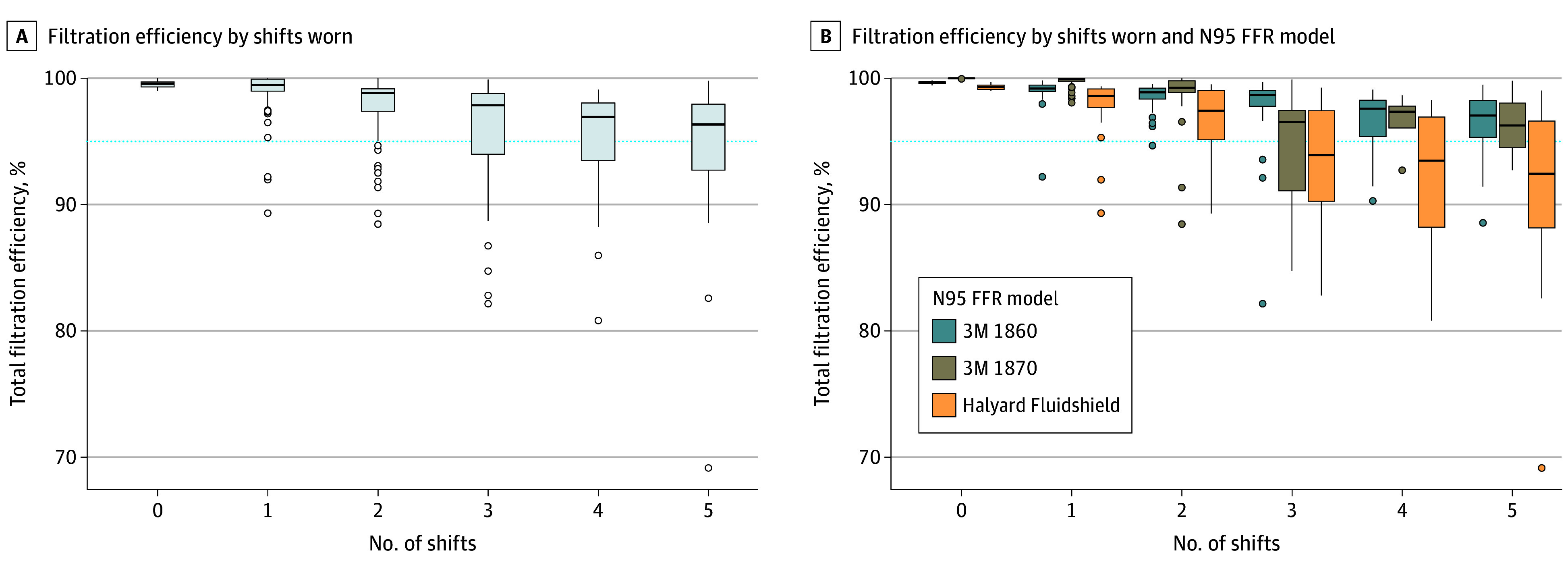
Boxplot of Percentage Filtration Efficiency by Shifts Worn and by N95s Model The dotted line indicates 95% filtration efficiency; circles, outliers, edges of boxes, upper and lower quartiles; center line in boxes, median value; whiskers, the minimum and maximum values.

**Table.** Filtration Efficiency and Proportion Below 95% by Shifts Worn, Stratified by N95s Model

Respirator model and shifts worn	No.	Filtration efficiency		
		Median (IQR), %	Mean (SD), %	<95%, No. (%)^a^
**All models**				
0	30	99.6 (99.3-99.7)	99.5 (0.3)	0
1	170	99.5 (99.0-99.9)	99.2 (1.3)	3 (1.8)
2	89	98.8 (97.4-99.2)	97.8 (2.4)	9 (10.1)
3	52	97.9 (94.0-98.8)	96.0 (4.5)	15 (28.8)
4	29	96.9 (93.5-98.0)	95.2 (4.3)	10 (34.5)
5	25	96.3 (92.7-97.9)	94.2 (6.6)	10 (40.0)
**Respirator 1**				
0	10	99.7 (99.6-99.7)	99.7 (0.1)	0
1	48	99.2 (98.9-99.5)	99.0 (1.1)	1 (2.1)
2	39	98.9 (98.3-99.2)	98.6 (1.0)	1 (2.6)
3	30	98.7 (97.8-99.0)	97.6 (3.4)	3 (10.0)
4	16	97.6 (95.4-98.2)	96.5 (2.6)	3 (18.8)
5	15	97.1 (95.3-98.2)	96.1 (3.1)	4 (26.7)
**Respirator 2**				
0	5	100.0 (100.0-100.0)	100.0 (0)	0
1	86	99.9 (99.7-100.0)	99.8 (0.4)	0
2	19	99.2 (98.9-99.8)	98.3 (3.1)	2 (10.5)
3	8	96.5 (91.1-97.4)	94.3 (5.1)	3 (37.5)
4	4	97.3 (96.1-97.8)	96.5 (2.6)	1 (25.0)
5	2	96.3 (94.5-98.0)	96.3 (5.0)	1 (50.0)
**Respirator 3**				
0	15	99.3 (99.1-99.5)	99.3 (0.2)	0
1	36	98.6 (97.7-99.1)	98.0 (2.0)	2 (5.6)
2	31	97.4 (95.1-99.0)	96.7 (2.7)	6 (19.4)
3	14	93.9 (90.3-97.4)	93.4 (4.9)	9 (64.3)
4	9	93.4 (88.2-96.9)	92.1 (5.9)	6 (66.7)
5	8	92.4 (88.2-96.6)	89.9 (9.9)	5 (62.5)

^a^ No. and proportion of all N95s worn for 0, 1, 2, 3, 4, and 5 shifts that had a filtration efficiency below 95%.

## Discussion

We found the number of shifts of reuse and respirator model were associated with reduced FE. While associated with minimal reduction in FE after 1 shift, after 3 shifts almost one-third of respirators did not filter 95% of particles. This reduction in FE differed by filtering facepiece respirator model. Limitations included observational design; because this was an observational study conducted within a larger study of N95 fit failure, randomization to the exposure was not feasible. Also, respirator effectiveness depends heavily on use in clinical practice, and FE is just 1 factor that contributes to the protection filtering facepiece respirators provide. Adherence to user instructions and fit are other factors, and it is important to note that all N95s had failed fit testing or were deemed unsuitable for further wear.^2,6^ Our findings suggest that while FE is preserved in N95s after a single shift of wear, ongoing reuse is significantly associated with reduced filtration performance.

## Notes

Supplement 1. **eAppendix.** Filtration Efficiency Measurement Protocol

Supplement 2.
Reuse N95 Group Members

Supplement 3.
Data Sharing Statement
