# Supplementary material for: N95 Filtering Facepiece Respirator Reuse, Extended Use, and Filtration Efficiency
Source: JAMA Netw Open. 2024 Oct 29;7(10):e2441663. doi: 10.1001/jamanetworkopen.2024.41663 (PMC11581496; doi:10.1001/jamanetworkopen.2024.41663)
Supplement: Supplement 1. — eAppendix. Filtration Efficiency Measurement Protocol [file jamanetwopen-e2441663-s001.pdf]

Supplemental Online Content

Wang RC, Addo N, Degesys NF, et al. N95 filtering facepiece respirator reuse, extended use, and filtration efficiency. *JAMA Netw Open*. 2024;7(10):e2441663. doi:10.1001/jamanetworkopen.2024.41663

eAppendix. Filtration Efficiency Measurement Protocol

This supplemental material has been provided by the authors to give readers additional information about their work.

© 2024 Wang RC et al. *JAMA Network Open.*

Supplementary Material

eAppendix. Filtration efficiency measurement protocol

Each participant was asked to wear an N95 FFR until fit failure occurred, the N95 FFR became soiled or deformed, or five clinical shifts had elapsed. N95 FFRs were then collected and shipped to Applied Research Associates (ARA, Panama City FLA) for filtration efficiency assessment.

A TSI 8130A automated filter tester (TSI Inc, St Paul, MN) was used to measure pressure drop and percent filter penetration. The TSI 8130A filter tester delivers a solid polydisperse sodium chloride (NaCl) aerosol that meets the particle size distribution criteria set forth in 42 CFR 84 Subpart K, Section 84.174 for NIOSH certification. All filtration tests were conducted at room temperature with a continuous airflow of 85 ± 2 L/min in accordance with the NIOSH certification criteria for challenging N95 filtering facepiece respirators.

Prior to filtration testing, N95 FFRs were treated with vaporized hydrogen peroxide (VHP) in a Cell-IQ™ series CO_2 Incubator with VHP capabilities (Model # MCO-170AICUVHL-PA, PHC Corporation of North America, Wood Dale, IL). Spordex test strips with 10^6 Geobacillus stearothermophilus spores (NA039; Steris Corporation, Mentor OH) were used as a biological indicator to confirm the efficacy of the VHP treatment. Once decontaminated, N95 FFRs were sealed in an upright position to a flat acrylic plate with a ~38-mm-diameter central opening using beeswax (eBeeHoney, Cat# 1005) and affixed to a test box compatible with the TSI 8130A automated filter tester. For N95 FFRs demonstrating a penetration value above the baseline penetration, ARA added additional sealant and repeated the test. This process was repeated a maximum of three times. For N95 FFRs tested more than once, the lowest penetration value was used for data analysis.

© 2024 Wang RC et al. *JAMA Network Open.*
