# Supplementary material for: N95 Filtering Facepiece Respirator Reuse, Extended Use, and Filtration Efficiency
Source: JAMA Netw Open. 2024 Oct 29;7(10):e2441663. doi: 10.1001/jamanetworkopen.2024.41663 (PMC11581496; doi:10.1001/jamanetworkopen.2024.41663)
Supplement: Supplement 3. — Data Sharing Statement [file jamanetwopen-e2441663-s003.pdf]

# Data Sharing Statement

Wang. N95 Filtering Facepiece Respirator Reuse, Extended Use, and Filtration Efficiency. *JAMA Netw Open*. Published October 29, 2024. doi:10.1001/jamanetworkopen.2024.41663

**Data**
**Data available:** Yes
**Data types:** Deidentified participant data, Data (not involving human participants), Data dictionary
**How to access data:** anna.harris@ucsf.edu OR ralph.wang@ucsf.edu
**When available:** With publication

**Supporting Documents**
**Document types:** None

**Additional Information**
**Who can access the data:** researchers whose proposed use of data has been approved
**Types of analyses:** for projects approved by UCSF and CDC
**Mechanisms of data availability:** after approval of proposal with signed data access agreement
**Any additional restrictions:** subject to CDC and UCSF discretion
